# Supplementary material for: The Evolution and Characterization of the RNA Interference Pathways in Lophotrochozoa
Source: Genome Biol Evol. 2024 May 7;16(5):evae098. doi: 10.1093/gbe/evae098 (PMC11114477; doi:10.1093/gbe/evae098)
Supplement: evae098_Supplementary_Data [file evae098_supplementary_data.zip › Supplementary_materials.zip!/selected_miRNAs/NoGe_selected_miRNAs/novel_miRNA_families_pdf_files/NMRB01000030.1_Notospermus_geniculatus_strain_Ushimado_scaffold30,_whole_genome_shotgun_sequence_1262.pdf]

Provisional ID	:	NMRB01000030.1_Notospermus_geniculatus_strain_Ushimado_scaffold30,_whole_genome_shotgun_sequence_1262
Score total	:	1738
Score for star read(s)	:	3.9
Score for read counts	:	1732.5
Score for mfe	:	2.2
Score for randfold	:	0
Score for cons. seed	:	-0.6
Total read count	:	3410
Mature read count	:	2408
Loop read count	:	0
Star read count	:	1002

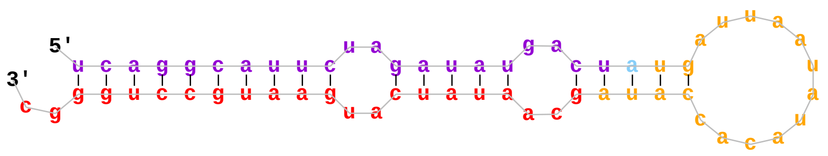

Genome coordinates: NMRB01000030.1 833517-833579

pre-miRNA sequence:
TCAGGCATTCTAGATATGACTTCAGGCATTCTAGATATGACTATGATTAATATACACCATAGCAATATCATGAATGCCTGGGC

Mature sequence: GCAATATCATGAATGCCTGGGC

BLAST results for the pre-miRNA sequence against the *Lineus longissimus* genome assembly:

**Scaffold**	**Start**	**Stop**	**Identity**	**e-value**
OU342998.1 1	9077010	9077071	95.161	2.36e-20

```
Nge_novel_miRNA    TCAGGCATTCTAGATATGACTATGATTAATATACACCATAGCAATATCATGAATGCCTGGGC
Llo_genome         TCAGGCATTCTAGATATGACTATGATTGATAAACGCCATAGCAATATCATGAATGCCTGGGC
```
